# Glutamatergic synaptic plasticity in the mesocorticolimbic system in addiction

**DOI:** 10.3389/fncel.2014.00466

**Published:** 2015-01-20

**Authors:** Aile N. van Huijstee, Huibert D. Mansvelder

**Affiliations:** Department of Integrative Neurophysiology, Center for Neurogenomics and Cognitive Research, Neuroscience Campus Amsterdam, VU University AmsterdamAmsterdam, Netherlands

**Keywords:** addiction, drugs of abuse, synaptic plasticity, glutamate, dopamine, ventral tegmental area, nucleus accumbens, prefrontal cortex

## Abstract

Addictive drugs remodel the brain’s reward circuitry, the mesocorticolimbic dopamine (DA) system, by inducing widespread adaptations of glutamatergic synapses. This drug-induced synaptic plasticity is thought to contribute to both the development and the persistence of addiction. This review highlights the synaptic modifications that are induced by *in vivo* exposure to addictive drugs and describes how these drug-induced synaptic changes may contribute to the different components of addictive behavior, such as compulsive drug use despite negative consequences and relapse. Initially, exposure to an addictive drug induces synaptic changes in the ventral tegmental area (VTA). This drug-induced synaptic potentiation in the VTA subsequently triggers synaptic changes in downstream areas of the mesocorticolimbic system, such as the nucleus accumbens (NAc) and the prefrontal cortex (PFC), with further drug exposure. These glutamatergic synaptic alterations are then thought to mediate many of the behavioral symptoms that characterize addiction. The later stages of glutamatergic synaptic plasticity in the NAc and in particular in the PFC play a role in maintaining addiction and drive relapse to drug-taking induced by drug-associated cues. Remodeling of PFC glutamatergic circuits can persist into adulthood, causing a lasting vulnerability to relapse. We will discuss how these neurobiological changes produced by drugs of abuse may provide novel targets for potential treatment strategies for addiction.

## Introduction

For a long time, addiction was not perceived as a disease, but as a personal choice. Therefore, little effort went into finding appropriate treatment strategies for drug-dependent individuals. In the last decades people have come to realize that addictive drugs cause pathological changes in brain function, and that addiction is a chronic medical disorder. Addiction, or “substance use disorder” as described in the DSM-5 (American Psychiatric Association, 2013), is characterized by compulsive drug use despite negative consequences and high rates of relapse. Globally, an estimated 27 million people are problem drug users of opiates, cocaine, cannabis or amphetamines and 1 in every 100 deaths among adults is attributed to illicit drug use (United Nations Office on Drugs and Crime, 2014). Furthermore, WHO estimates that the social cost of illicit substance use was around 2% of the gross domestic product in 2004 in countries that have measured it (World Health Organisation, 2008). Most of these costs were associated with drug-related crime. Although the European drug landscape has remained relatively stable in recent years, drug use in Europe remains high by historical standards (EMCDDA, 2013). On top of that, the costs and mortality as a direct consequence of the use of socially accepted drugs, such as tobacco smoking and drinking alcohol, are enormous. According to the WHO, tobacco kills nearly 6 million people each year and 3.3 million people die every year due to harmful use of alcohol. This does not take into account the causal relationships between both harmful use of alcohol and tobacco smoking and a range of mental and behavioral disorders (Volkow and Li, 2005). All in all, the global burden of addiction is immense. Unfortunately, current treatments for drug addiction are still relatively ineffective. To improve treatment strategies, it is important to better understand the neurobiological changes that underlie addiction.

Although the brain circuitry underlying addiction is complex, it is well established that the brain’s reward circuitry, or more specifically, the mesocorticolimbic dopamine (DA) system, plays an important role. The mesocorticolimbic system consists of the ventral tegmental area (VTA) and the brain regions that are innervated by projections from the VTA, such as the nucleus accumbens (NAc), the prefrontal cortex (PFC), the amygdala, and the hippocampus (Swanson, 1982). The mesocorticolimbic system is crucial for reward and reinforcement processing, motivation, and goal-directed behavior (Schultz, 1998; Wise, 2004). All addictive drugs act on the mesocorticolimbic system, increasing DA levels in this system (Di Chiara and Imperato, 1988). Different types of drugs increase mesocorticolimbic DA levels through distinct cellular mechanisms. Nicotine increases DA levels by directly stimulating VTA DA neurons through α4β2-containing nicotinic receptors (Maskos et al., 2005). Several other drugs, such as benzodiazepines, opioids and cannabinoids, exert their effect by inhibiting GABAergic interneurons in the VTA, thereby reducing inhibition on the DA neurons (Johnson and North, 1992; Szabo et al., 2002; Tan et al., 2010). Psychostimulants, such as cocaine and amphetamine, increase extracellular DA levels by interacting with the DA transporter, thereby inhibiting DA reuptake (Williams and Galli, 2006). The increase in mesocorticolimbic DA levels mediates the acute reinforcing effects of addictive drugs. It does however not explain the long-lasting behavioral abnormalities that are seen in addiction, since these are still present once the drugs have been cleared from the body and the DA levels have returned to normal. The development and expression of addictive behaviors are caused by lasting drug-induced neuroadaptations in the mesocorticolimbic system.

Accumulating evidence indicates that drugs indeed induce long-lasting changes in the brain. More specifically, drugs of abuse modify synaptic transmission in the mesocorticolimbic system. This phenomenon is called drug-induced synaptic plasticity (Lüscher and Malenka, 2011). This review focuses on synaptic plasticity induced by *in vivo* exposure to drugs of abuse. Although many types of synapses are modified by addictive drugs, addictive drugs are thought to mainly alter glutamatergic transmission (Lüscher, 2013). Furthermore, drug-induced synaptic plasticity of glutamatergic transmission has been extensively studied. We will highlight recent findings on drug-induced synaptic plasticity of glutamatergic transmission, with the aim to elucidate what the common mechanisms behind these synaptic adaptations are, and how these drug-induced synaptic changes contribute to the different aspects of addictive behavior.

## Early changes in synaptic transmission

### Synaptic plasticity in the ventral tegmental area (VTA)

Changes in synaptic transmission already occur after the first exposure to an addictive drug. A single *in vivo* exposure to an addictive drug induces an increase in synaptic strength at glutamatergic synapses onto DA neurons of the VTA. A single noncontingent injection of cocaine was administered *in vivo* to mice and rats, and 24 h later, excitatory postsynaptic currents (EPSCs) were recorded in dopaminergic neurons in midbrain slices of these animals to monitor changes in synaptic strength (Ungless et al., 2001). The ratio of AMPAR- vs. NMDAR-mediated EPSCs (AMPAR/NMDAR ratio) in VTA DA neurons was significantly increased. This increase can reflect an increase in AMPAR currents or a decrease in NMDAR currents or a combination of both. In fact, increased AMPAR currents and decreased NMDAR currents both occur and cause the increased AMPAR/NMDAR ratio after exposure to an addictive drug. Ungless et al. (2001) demonstrated that AMPAR transmission was enhanced at glutamatergic synapses onto DA neurons of the VTA, since both the amplitude and frequency of AMPAR-mediated miniature EPSCs (mEPSCs) were significantly increased after *in vivo* exposure to cocaine. This was further supported by the finding that exogenously applied AMPA to VTA DA neurons led to larger AMPA-induced currents in slices from mice that had received a cocaine injection than in slices from mice that were injected with saline. Importantly, the cocaine-induced long-term potentiation of AMPAR-mediated currents was demonstrated to be specific for DA neurons of the VTA, since no potentiation was found in the hippocampus or in GABA neurons in the VTA. Cocaine-induced increases in the AMPAR/NMDAR ratio did not occur when cocaine was co-administered with an NMDAR antagonist, showing that the increase in the AMPAR/NMDAR ratio depends on NMDAR activation. Thus, a single exposure to cocaine induces synaptic plasticity at excitatory synapses on VTA DA neurons that is similar to NMDAR-dependent long-term potentiation (LTP). In line with this, drug-induced synaptic plasticity occludes subsequent LTP, suggesting that these two types of plasticity share underlying mechanisms (Ungless et al., 2001; Liu et al., 2005; Argilli et al., 2008; Luu and Malenka, 2008).

After the discovery of* in vivo* cocaine-induced plasticity, it was investigated whether other drugs of abuse elicit the same changes in excitatory transmission in the VTA. From *in vitro* studies it was already known that application of even a low dose of nicotine induces LTP at excitatory synapses onto VTA DA neurons (Mansvelder and McGehee, 2000), supporting this idea. Indeed, all addictive drugs tested to date (among which morphine, nicotine, benzodiazepines and ethanol) induce a potentiation of AMPAR transmission in VTA DA neurons 24 h after *in vivo* administration of a single dose (Saal et al., 2003; Tan et al., 2010). AMPAR/NMDAR ratios were not increased after *in vivo* administration of the non-addictive psychoactive drugs fluoxetine and carbamazepine (Saal et al., 2003). The fact that different drug classes, with different molecular mechanisms of action, induce a similar type of synaptic plasticity in VTA DA neurons was a first indication that this form of synaptic plasticity might be related to the addictive properties of these drugs.

Further research into the mechanisms underlying the increased AMPAR/NMDAR ratios showed that NMDAR-activation needed for the drug-induced synaptic plasticity occurs through stimulation of DA D_5_ receptors, since no cocaine-induced potentiation was found after application of a D_1_/D_5_ receptor antagonist or in D_5_ receptor knockout mice (Argilli et al., 2008). Nicotine-induced synaptic plasticity *in vitro* also depends on DA D_5_ receptors (Mao et al., 2011). The enhanced AMPAR transmission is caused by the insertion of GluA2-lacking AMPARs into the synapse following *in vivo* exposure to addictive drugs (Bellone and Lüscher, 2006; Argilli et al., 2008). Early work by Fitzgerald et al. (1996) already provided an indication that drug-induced plasticity at glutamatergic synapses onto DA neurons of the VTA involved a switch to GluA2-lacking AMPARs. Expression of GluA1 subunits, but not GluA2 subunits, was increased in VTA DA neurons after exposure to cocaine (Fitzgerald et al., 1996). To find more direct evidence of a switch to GluA2-lacking AMPARs, Bellone and Lüscher (2006) made use of the distinct biophysical characteristics of GluA2-lacking AMPARs. GluA2-lacking AMPARs are Ca^2+^-permeable, have greater conductance, are inwardly rectifying and are blocked by polyamines (Washburn and Dingledine, 1996; Isaac et al., 2007). The rectification of AMPAR-mediated EPSCs was increased following cocaine administration, and administration of the polyamine toxin Joro spider toxin (JST) partially blocked AMPAR-mediated EPSCs in cocaine-treated mice, confirming the insertion of GluA2-lacking AMPARs (Bellone and Lüscher, 2006). These receptors could be inserted on top of the existing pool of GluA2-containing AMPARs or they could replace GluA2-containing receptors, keeping the total number of AMPARs at the synapse constant. Immunogold labeling of GluA2 subunits showed that the cytoplasmic pool of GluA2-containing AMPARs was enhanced after exposure to cocaine, while GluA2 labeling at the synapse was reduced (Mameli et al., 2007). Thus, exposure to addictive drugs causes an exchange of GluA2-containing for GluA2-lacking AMPARs, which potentiates AMPAR transmission because of the higher single-channel conductance of the GluA2-lacking AMPARs.

Originally, studies that reported a drug-induced potentiation of AMPAR-mediated transmission in VTA DA neurons were performed 24 h after drug administration, but what is the actual time course of synapse strengthening following drug exposure? Argilli et al. (2008) found that synaptic plasticity occurred more rapidly after administration, since AMPAR potentiation was found to be present within 3 h (Argilli et al., 2008). Cocaine-induced synaptic plasticity is transient, since synaptic potentiation was still observed after 5 days, but not after 10 days (Ungless et al., 2001). Since transmission at glutamatergic synapses onto VTA DA neurons is normalized after approximately a week, a process counteracting the drug-induced changes must be involved. Interestingly, cocaine-induced synaptic plasticity is reversed by activation of mGluR1 receptors in the VTA. More specifically, intraperitoneal injection of a positive modulator of mGluR1 reversed cocaine-induced AMPAR subunit redistribution (Bellone and Lüscher, 2006). Local disruption of mGluR1 function in neurons of the VTA on the other hand prolonged the cocaine-evoked potentiation in the VTA (Mameli et al., 2009). Thus, following exposure to drugs, glutamatergic synaptic transmission is normalized by mGluR-dependent LTD, which consists of a replacement of GluA2-lacking AMPARs with lower conductance GluA2-containing AMPARs (Mameli et al., 2007).

Thus far, enhanced AMPAR transmission has been discussed as a reason for the increased AMPAR/NMDAR ratios induced by *in vivo* drug exposure. However, the insertion of the GluA2-lacking AMPARs would actually decrease the AMPAR/NMDAR ratio at +40 mV, since GluA2-lacking AMPARs conduct poorly at positive membrane potentials due to polyamine block. Therefore, the increased AMPAR/NMDAR ratios must partly be caused by a decrease in NMDAR currents. Indeed, increased AMPAR/NMDAR ratios were also found to be due to a decrease in NMDAR transmission (Mameli et al., 2011). This decrease results from a NMDAR subunit composition switch. GluN2B to GluN2A ratios are increased following exposure to cocaine (Yuan et al., 2013). Furthermore, GluN3A-containing NMDARs, which have very low Ca^2+^ permeability, are inserted into the synapse after cocaine exposure (Yuan et al., 2013). Together, these findings suggest that cocaine drives the synaptic insertion of tri-heteromeric GluN1/GluN2B/GluN3A-containing NMDARs that replace GluN1/GluN2As and GluN1/GluN2A/ GluN2Bs. Both cocaine-induced changes in AMPAR transmission and cocaine-induced changes in NMDAR transmission were found to be dependent on insertion of GluN3A-containing NMDARs, since no cocaine-induced plasticity was present in GluN3A knockout mice or after injection of an adeno-associated viral vector expressing an anti-GluN3A short-hairpin RNA (Yuan et al., 2013). Finally, mGluR1 activation, which reverses cocaine-induced AMPAR subunit redistribution, also reversed NMDAR subunit redistribution (Yuan et al., 2013). Thus, the synaptic insertion of GluN3A-containing AMPARs appears to be essential for the expression of cocaine-induced plasticity.

In addition to glutamatergic inputs, VTA DA neurons also receive GABAergic inputs, from local interneurons and from projections from the NAc and the ventral pallidum (VP; Kalivas et al., 1993; Steffensen et al., 1998). These inhibitory synapses onto VTA DA neurons also undergo synaptic plasticity after exposure to addictive drugs. Morphine, cocaine and nicotine were all shown to impair long-term potentiation of GABAergic synapses onto VTA DA neurons, albeit with different time courses (Niehaus et al., 2010). On the other hand, GABAergic inputs to inhibitory interneurons in the VTA are potentiated after *in vivo* exposure to drugs, thereby disinhibiting DA neurons (Tan et al., 2010; Bocklisch et al., 2013). Together, the increased AMPAR/NMDAR ratio at glutamatergic synapses onto VTA DA neurons, the loss of LTP of GABAergic synapses onto VTA DA neurons, and the disinhibition of VTA DA neurons by potentiation of GABAergic synapses onto VTA interneurons are likely to enhance the excitability of VTA DA neurons. In the case of nicotine, GABAergic input onto VTA DA neurons is further decreased by desensitization of nicotinic acetylcholine receptors (nAChRs) on GABAergic neurons (Mansvelder et al., 2002). Since activation of nAChRs depolarizes GABAergic neurons, this desensitization leads to a depression of GABAergic transmission. This effect is the greatest in the subset of GABAergic neurons that are normally excited by endogenous cholinergic transmission. The depression of GABAergic inputs due to nAChR desensitization may contribute to an increase in VTA DA neuron excitability, although more complex interactions between GABA and DA neuron activity seem to play a role (Tolu et al., 2013).

Drug-induced synaptic plasticity in the VTA does not occur globally, affecting all VTA DA neurons equally. Recent findings have shown that drugs differentially affect different subpopulations of VTA DA neurons with projections to different target areas (Lammel et al., 2013). A single injection of cocaine selectively modified glutamatergic synapses on DA neurons projecting to the NAc shell but not synapses on DA neurons projecting to the mPFC (Lammel et al., 2011). Excitatory synapses on DA neurons projecting to the mPFC were however changed by an aversive stimulus, suggesting that rewarding and aversive stimuli affect different DA neuron subpopulations in the VTA. The DA neurons projecting to the NAc shell, which are affected by drugs, receive their input from the laterodorsal tegmentum, while the DA neurons projecting to the mPFC, which are affected by aversive stimuli, receive their input from the lateral habenula (Lammel et al., 2012).

Single *in vivo* exposures to drugs of abuse induce synaptic changes that are still present long after the drugs have been cleared from the body, at least 5 days. However, this is too short-lived to explain the long-lasting behavioral effects that are seen in addiction. Even after repeated cocaine administration (seven daily injections), the cocaine-induced increase in the AMPAR/NMDAR ratio in VTA DA neurons is transient and can no longer be observed ten days after the cessation of cocaine administration, just as the synaptic potentiation induced by a single injection (Borgland et al., 2004). However, in all these studies noncontingent drug administration was used. The effect of self-administration of addictive drugs is longer lasting than the effect of noncontingent drug administration. After 3 months of abstinence from cocaine self-administration, cocaine-induced synaptic potentiation was still present in VTA DA neurons in rats (Chen et al., 2008). This persistence of the drug-induced synaptic potentiation in VTA neurons after drug self-administration suggests that this phenomenon could be a fundamental factor driving the development of addictive behavior.

What are the direct behavioral changes that are induced by drug-induced synaptic plasticity in the VTA? Mice with a genetic deletion of the GluA1 subunit or the GluN1 subunit selectively in DA neurons lacked drug-induced plasticity in VTA DA neurons, but cocaine still induced normal conditioned place preference (CPP) and behavioral sensitization in these mice (Engblom et al., 2008). In another study, mice lacking the GluN1 subunit exclusively in DA neurons also showed normal behavioral sensitization, but CPP was abolished in these mice (Zweifel et al., 2008), in contrast with the results from Engblom et al. (2008). This discrepancy between the studies could be caused by differences in CPP protocols. All in all, evidence that the drug-induced synaptic plasticity in VTA DA neurons mediates short-term behavioral effects of drug exposure is very limited. However, the drug-induced synaptic plasticity in VTA DA neurons may be an important first step for late-stage addictive behaviors. In mice lacking GluN1 in DA neurons, reinstatement of drug seeking behavior in CPP was abolished (Engblom et al., 2008). Furthermore, the delayed enhancement of behavioral sensitization that occurs after prolonged cocaine withdrawal did not occur in GluN1 knockout mice (Zweifel et al., 2008). These late-stage behavioral changes are probably not direct consequences of the synaptic plasticity in the VTA, but are more likely caused by synaptic changes in other brain areas that are triggered by the synaptic plasticity in the VTA. Drug-induced synaptic plasticity in the VTA leads to subsequent synaptic plasticity in other brain areas, which is more important for the behavioral changes seen in addiction than the synaptic plasticity in the VTA itself (see below). In the VTA, drugs of abuse alter the rules for activity-dependent plasticity and thereby enable subsequent downstream changes throughout the mesocorticolimbic circuitry (Creed and Lüscher, 2013). These changes in downstream brain regions, in particular in the NAc and the PFC, are then thought to be associated with the long-lasting behavioral abnormalities that are seen in addiction.

The capacity of the drug-induced synaptic plasticity in the VTA to invert the rules for activity-dependent plasticity relies on the redistribution of AMPAR and NMDAR subunits (Yuan et al., 2013). Normally, LTP is NMDAR-dependent and is induced when presynaptic glutamate release coincides with depolarization of the postsynaptic membrane, because the Mg^2+^-block of the NMDARs is relieved at depolarized membrane potentials, allowing Ca^2+^ entry. However, cocaine induces a switch of NMDAR subunit composition, creating NMDARs that have very low Ca^2+^ permeability (Yuan et al., 2013). Therefore, activation of NMDARs no longer induces LTP. On the other hand, cocaine drives the synaptic insertion of GluA2-lacking AMPARs, which are Ca^2+^-permeable. Therefore, after cocaine exposure, a form of LTP can be induced in VTA neurons that relies on calcium entry through AMPARs and is independent of NMDARs (Mameli et al., 2011). However, GluA2-lacking AMPARs conduct poorly at positive membrane potentials, due to polyamine block at positive membrane potentials. The greater the hyperpolarization, the more readily GluA2-lacking AMPARs conduct calcium. Therefore, cocaine causes the rules of activity-dependent synaptic potentiation to change, since the induction of LTP now requires a pairing of presynaptic activity with hyperpolarization of the postsynaptic cell instead of depolarization (Mameli et al., 2011). Whether this holds true for all drugs of abuse remains to be tested.

As mentioned above, glutamatergic synaptic plasticity in the VTA triggers subsequent synaptic plasticity in other parts of the mesocorticolimbic system. Synaptic changes occur first in the VTA before synaptic plasticity in other regions of the mesocorticolimbic system. In addition, changes in the VTA occur after a single exposure, whereas plasticity in downstream areas generally requires multiple drug exposures (Kourrich et al., 2007), although it needs to be noted that Mato et al. (2004) showed that in mice, a single *in vivo* administration of THC altered synaptic plasticity in the NAc (Mato et al., 2004). Furthermore, cocaine-evoked synaptic plasticity in the NAc only occurs if the synaptic plasticity in the VTA is persistent (Mameli et al., 2009). Reversing synaptic plasticity in the VTA by intraperitoneal injection of a positive modulator of mGluR1 prevented synaptic plasticity in the NAc (Mameli et al., 2009). On the other hand, after local disruption of mGluR1 function in VTA neurons, which prolongs drug-evoked potentiation in the VTA, a single injection of cocaine was sufficient to induce synaptic plasticity in the NAc, which is not normally the case. These findings suggest a hierarchical organization of drug-induced synaptic plasticity, with the VTA as first station of plasticity, followed by glutamatergic synaptic plasticity in downstream areas of the mesocorticolimbic system. Mechanisms underlying these later stages of plasticity are discussed in the following section.

## Later stages of synaptic remodeling

### Drug-induced synaptic plasticity in the nucleus accumbens

The principal neurons of the NAc, the GABAergic medium spiny neurons (MSNs), receive glutamatergic inputs from cortical and limbic brain regions, including the PFC and the amygdala (Groenewegen et al., 1999). Results from several studies indicate that glutamatergic transmission in the NAc plays an important role in drug seeking. Microinjection of AMPA into the NAc elicits significant reinstatement of cocaine-seeking behavior, while microinjection of an AMPAR antagonist into the NAc prevents reinstatement of cocaine seeking (Cornish and Kalivas, 2000; Ping et al., 2008). Furthermore, activity within the NAc is thought to mediate behavioral sensitization (Koya et al., 2009, 2012). Since glutamatergic signaling in the NAc plays an important role in addictive behavior, synaptic plasticity at glutamatergic synapses onto NAc MSNs is likely to be involved in addiction.

In contrast to the VTA, a single cocaine injection is not sufficient to induce synaptic plasticity in the NAc (Kourrich et al., 2007). Only after repeated treatment with cocaine, changes in synaptic strength occur at glutamatergic synapses on NAc MSNs. The direction of this synaptic plasticity is dependent on the duration of drug withdrawal (Kourrich et al., 2007). Kourrich et al. (2007) administered five once-daily cocaine injections to male mice and assessed the AMPAR/NMDAR ratio in NAc shell neurons both 24 h and 10–14 days after the last injection. During early withdrawal, 24 h after the last injection, a significant decrease in AMPAR/NMDAR ratios was found in NAc shell neurons of cocaine-treated mice. However, after a drug-free period of 10–14 days, AMPAR/NMDAR ratios were increased in NAc shell neurons. Furthermore, a single drug re-exposure led to a decrease in AMPAR/NMDAR ratios 1 day later, abruptly reversing the synaptic potentiation. Synaptic depression following an additional cocaine exposure after a drug-free period was also described in earlier studies (Thomas et al., 2001; Brebner et al., 2005). Thomas et al. (2001) furthermore showed that the decrease in the AMPAR/NMDAR ratio reflects a reduction in AMPAR transmission.

To determine the cause of increased AMPAR/NMDAR ratios in NAc shell neurons after prolonged withdrawal, Kourrich et al. (2007) recorded AMPAR-mediated mEPSCs. The amplitude and frequency of AMPAR-mediated mEPSCs were significantly increased in cocaine-treated mice (Kourrich et al., 2007), suggesting a potentiation of AMPAR-mediated synaptic transmission after prolonged withdrawal. These results match findings by Boudreau and Wolf (Boudreau and Wolf, 2005), who found increased cell surface expression of AMPAR subunits in the NAc of rats after 21 days of withdrawal. Although the exact mechanisms underlying this drug-induced synaptic potentiation in the NAc shell after prolonged withdrawal are unknown, it is known that the synaptic potentiation is dependent on activation of the extracellular signal-regulated kinase (ERK) pathway by way of ERK phosphorylation (Pascoli et al., 2012). The increased frequency of AMPAR mEPSCs, combined with the finding that the paired-pulse ratio (a measure for presynaptic function) was unchanged, suggests that repeated exposure to cocaine may lead to the formation of new synapses on NAc MSNs. This is supported by the finding that the number of dendritic spines on NAc MSNs was increased after repeated exposure to cocaine, amphetamine and nicotine (Robinson and Kolb, 2004). Thus, repeated cocaine exposure produces synaptic potentiation in the NAc shell that develops during drug withdrawal, possibly due to formation of new synapses, which is followed by an abrupt synaptic depression upon a single re-exposure. However, both after prolonged withdrawal and after a single drug re-exposure, NMDAR transmission was unaltered (Kourrich et al., 2007). Furthermore, no evidence for a change in the presence of GluA2-lacking AMPARs at the synapse was found, since the rectification index of AMPAR-mediated EPSCs was unchanged in cocaine-treated mice after prolonged withdrawal and after additional cocaine exposure.

These studies used noncontingent drug administration to elicit synaptic plasticity. Following cocaine self-administration, synaptic depression of excitatory transmission was found in the NAc shell during early withdrawal from cocaine (Schramm-Sapyta et al., 2006), which was followed by increased AMPAR transmission after prolonged withdrawal (Conrad et al., 2008). However, in contrast to the findings by Kourrich et al. (2007), this increase was found to be due to the synaptic insertion of GluA2-lacking AMPARs. Both surface and intracellular GluA1 levels were significantly increased in the NAc after 45 days of withdrawal, and inward rectification of AMPAR-mediated EPSCs was significantly increased in the NAc core of cocaine-treated rats (Conrad et al., 2008). GluA2 levels were not changed, suggesting that GluA2-lacking AMPARs are inserted on top of the existing pool of GluA2-containing AMPARs. Since Kourrich et al., assessed synaptic changes after 10–14 days withdrawal, whereas Conrad et al. checked synaptic changes after 42–47 days withdrawal, it is possible that the synaptic insertion of GluA2-lacking AMPARs in the NAc only occurs after a sustained period of withdrawal. This is in accordance with the finding that NAc GluA1 levels were only slightly increased after 21 days of withdrawal, indicating that GluA1 levels increase gradually after withdrawal (Conrad et al., 2008). The study by Mameli et al. (2009) supports the idea that GluA2-lacking AMPARs are inserted into synapses after extended withdrawal from repeated cocaine exposure. An increased rectification index was found in NAc MSNs after 35 days of withdrawal after both noncontingent cocaine administration and self-administration of cocaine (Mameli et al., 2009). Thus, synaptic AMPAR subunit levels in NAc MSNs are changing for several weeks following cocaine exposure.

Both Thomas et al. (2001) and Kourrich et al. (2007) found a significant decrease in AMPAR/NMDAR ratios in NAc shell neurons of cocaine-treated mice during early withdrawal, which was attributed to a decrease in AMPAR transmission (Thomas et al., 2001). However, a decreased AMPAR/NMDAR ratio may also be caused by the generation of silent synapses. Silent synapses are glutamatergic synapses that contain NMDARs, but no functional AMPARs (Isaac et al., 1995). Thomas et al. (2001) did not find any cocaine-induced changes in NMDAR transmission, but this does not exclude the possibility that the number of silent synapses is increased by cocaine exposure. Indeed, following repeated noncontingent cocaine exposure the number of silent synapses was increased in NAc shell MSNs (Huang et al., 2009). These new silent synapses were gradually generated during cocaine exposure, producing a significant increase in the percentage of silent synapses from the third day of the cocaine treatment. Cocaine-induced generation of silent synapses was mediated by membrane insertion of new GluN2B-containing NMDARs (Huang et al., 2009). Surface and total levels of GluN2B, but not GluN2A, were increased, combined with an increased surface level of obligatory GluN1 subunits. Furthermore, after selectively inhibiting GluN2B-containing NMDARs, no increase in silent synapses could be detected. During withdrawal, the number of silent synapses declined again (Huang et al., 2009). However, this does not necessarily mean that the generated synapses disappeared again. It could also mean that newly generated silent synapses were unsilenced by synaptic insertion of (GluA2-lacking) AMPARs. This might be one of the mechanisms behind the increased cell surface expression of AMPAR subunits and the increased AMPAR/NMDAR ratio in NAc shell neurons after prolonged withdrawal. Thus, the observed changes in the AMPAR/NMDAR ratio and the observed changes in the number of silent synapses might go hand in hand. Furthermore, the generation of silent synapses may facilitate subsequent synaptic plasticity.

The behavioral relevance of decreases in AMPAR/NMDAR ratios during early withdrawal is not yet clear. It has been proposed that the drug-induced synaptic depression might cause NAc shell neurons to be less sensitive to natural rewarding stimuli, which could result in feelings of anhedonia and dysphoria (Van den Oever et al., 2012). This might increase drug craving. The behavioral consequence of synaptic depression following a single drug re-exposure is thought to be the acute expression of behavioral sensitization (Brebner et al., 2005). Blocking the induction of synaptic depression by intra-NAc infusion of a membrane permeable peptide that disrupts endocytosis of GluA2 prevented increased locomotor activity induced by amphetamine re-exposure.

The gradual potentiation of AMPAR transmission during prolonged withdrawal may mediate “incubation of drug craving”, the phenomenon that cue-induced craving and drug seeking increase progressively over the first months after withdrawal from abused drugs (Grimm et al., 2001). Potentiation of AMPAR transmission enhances the responsiveness of NAc MSNS to drug-associated cues, leading to enhanced cue-induced drug craving and drug seeking. Cue-induced cocaine-seeking after prolonged withdrawal from cocaine self-administration was decreased by injecting a selective blocker of GluA2-lacking AMPARs into the NAc (Conrad et al., 2008). Furthermore, internalization of GluA2-lacking AMPARs at amygdala-to-NAc synapses by *in vivo* optogenetic stimulation after prolonged withdrawal decreased incubation of cocaine craving (Lee et al., 2013). The effect of this internalization was the re-silencing of some of the unsilenced synapses, suggesting that the maturation of silent synapses also contributes to incubation of cocaine craving.

The described drug-induced synaptic changes in the NAc led Bellone and Lüscher (2012) and Dong and Nestler (2014) to propose a hypothesis of rejuvenation of synaptic transmission during cocaine addiction, creating types of synapses that are normally associated with early brain development (Bellone and Lüscher, 2012; Dong and Nestler, 2014). Examples are the generation of silent synapses and the synaptic insertion of GluA2-lacking AMPARs. Exposure to cocaine reopens a critical period of synapse development in the mesocorticolimbic system. Younger synapses have a higher ability to undergo long-lasting experience-dependent plastic changes. This is thought to explain how drugs can elicit such strong and unusually long-lasting plastic changes, leading to equally strong and durable drug-associated memories (Dong and Nestler, 2014). The rejuvenation hypothesis also extends to synaptic changes in the VTA, such as the re-emergence of GluN3A-containing NMDARs. The fact that younger synapses have a higher ability to undergo experience-dependent plastic changes might also explain why younger people are more vulnerable to addiction.

Glutamatergic synaptic plasticity does not only occur during drug exposure and withdrawal. It also occurs during cue-induced relapse to drug seeking. The presentation of cocaine-associated cues in the absence of cocaine was found to elicit rapid increases in both the dendritic spine size and the AMPAR/NMDAR ratio in the NAc core of rats that had a history of cocaine self-administration (Gipson et al., 2013a). These increases were already present 15 min after the presentation of the cocaine-associated cue and the increase of the AMPAR/NMDAR ratio and the spine head diameter at 15 min were positively correlated with the intensity of drug seeking. This suggests that the rapid cue-induced synaptic potentiation in NAc core MSNs might be a mechanism that triggers relapse. Two hours after initiating cue-induced reinstatement, the dendritic spine size and the AMPAR/NMDAR ratio had returned to pre-relapse levels. Activity in the prelimbic cortex (PL), a subregion of the medial PFC (mPFC) that sends glutamatergic projections to the NAc core, was shown to be crucially involved, since microinjection of GABAR agonists into the PL prevented both cue-induced synaptic changes and drug seeking (Gipson et al., 2013a). Whether cue-induced activity of glutamatergic PL-to-NAc synapses is sufficient to trigger actual relapse, or whether cue-induced synaptic changes are necessary as well for relapse remains to be determined. However, a recent study argues for a causal link between cocaine-induced plasticity of glutamatergic mPFC-to-NAc synapses and relapse (Pascoli et al., 2014).

The impact of cue-induced activity at glutamatergic PL-to-NAc synapses is enhanced by the reduction in basal extracellular glutamate levels in the NAc associated with withdrawal from addictive drugs (Gipson et al., 2014). Basal extracellular glutamate levels in the NAc are reduced after both contingent and noncontingent cocaine and nicotine exposure due to downregulated activity of the glial cystine–glutamate exchanger, which transports glutamate to the extracellular space (Kalivas, 2009). This results in decreased glutamate tone on presynaptic inhibitory mGluR2 and mGluR3 receptors, which in turn decreases inhibition of presynaptic glutamate release (Gipson et al., 2014). This leads to an increased synaptic release of glutamate in response to drug-associated cues. Furthermore, the released glutamate is not efficiently removed from the synapse, because the expression of the glial glutamate transporter 1 (GLT-1), which mediates glutamate uptake, is diminished following exposure to drugs (Knackstedt et al., 2010; Shen et al., 2014). Thus, drug-associated cues give rise to excessive amounts of glutamate in NAc glutamatergic synapses, which may explain why these cues can have such a strong behavioral effect.

In addition to directly modifying synaptic strength in the NAc, drugs of abuse are also thought to alter the capacity of NAc synapses to undergo subsequent synaptic plasticity. Cocaine addiction is associated with an impairment of NMDAR-dependent LTP and LTD at glutamatergic synapses in the NAc core (Martin et al., 2006; Moussawi et al., 2009; Kasanetz et al., 2010). LTD was impaired in the NAc core, but not in the NAc shell, after 21 days of abstinence in rats that had self-administered cocaine (Martin et al., 2006). Drug-induced impairments in synaptic plasticity may contribute to inflexible, compulsive behaviors that are characteristic of addiction and might explain drug consumption despite negative health consequences in humans. The relevance of impaired NMDAR-dependent LTD with regard to cocaine addiction was emphasized by the finding that only cocaine self-administering rats that developed addiction-like behaviors showed persistently impaired LTD, while LTD was recovered in animals that maintained a controlled drug intake (Kasanetz et al., 2010). Similar results were found after self-administration of ethanol. NMDAR-dependent LTD was significantly impaired after protracted withdrawal in ethanol-sensitized mice, but not in ethanol-treated mice that failed to develop this behavioral adaptation (Abrahao et al., 2013). D-serine, a co-agonist of the NMDAR, might play a role in the drug-induced impairment in NMDAR-dependent synaptic plasticity (D’Ascenzo et al., 2014). D-serine is essential for NMDAR-dependent LTP and LTD in the NAc core and D-serine levels are reduced in the NAc core of cocaine-treated rats (Curcio et al., 2013). In slices from cocaine-treated rats, perfusion with D-serine fully restored LTP and LTD induction. Thus, cocaine-induced deficits in NMDAR-dependent synaptic plasticity in the NAc are due, at least partially, to reduced D-serine levels.

As in the VTA, there is some evidence that drugs do not only cause global synaptic changes in the NAc, but can also differentially affect different subpopulations of MSNs (Wolf and Ferrario, 2010; Creed and Lüscher, 2013). Synaptic potentiation in the NAc shell that develops during drug withdrawal is thought to selectively occur at synapses onto D1-receptor-expressing MSNs, and not at synapses onto D2-receptor-expressing MSNs (Pascoli et al., 2012). Recently, it was found that this synaptic potentiation at synapses onto D1-receptor-expressing MSNs is dependent on the formation of D1R/GluN1 complexes (Cahill et al., 2014). Blocking D1R/GluN1 association, while preserving individual D1R and NMDAR-dependent signaling, prevented ERK activation, which is required for the synaptic potentiation.

### Synaptic plasticity in the medial prefrontal cortex

Another main target of DA projections originating in the VTA is the medial prefrontal cortex (mPFC). The pyramidal neurons of the mPFC receive glutamatergic projections from many different brain areas, including the basolateral nucleus of the amygdala (BLA), and send glutamatergic projections to the VTA, the NAc and back to the BLA (Gabbott et al., 2005; Hoover and Vertes, 2007; Van den Oever et al., 2010). Addiction is associated with decreased basal PFC neuronal activity, or “hypofrontality” (Volkow et al., 2003), which can also be seen in rats after repeated cocaine self-administration (Sun and Rebec, 2006). The mPFC can be divided into a dorsal (including the PL) and a ventral part (including the infralimbic cortex) that have different anatomical and functional characteristics (Van den Oever et al., 2012). Anatomically, the dorsal mPFC is thought to predominantly innervate the NAc core, while the ventral mPFC sends projections to the NAc shell (Heidbreder and Groenewegen, 2003). The dorsal and ventral mPFC are also thought to play different roles in addiction. Activity in the dorsal mPFC is thought to initiate drug seeking and is crucial for cue-induced relapse to drug seeking (Gipson et al., 2013b). On the other hand, activity in the ventral mPFC can suppress drug-seeking responses (Peters et al., 2008; LaLumiere et al., 2012). As a whole, the mPFC is therefore thought to play an important role in the control of relapse behavior.

Only few studies investigating drug-evoked synaptic plasticity in the mPFC have been performed. Therefore, we have only limited knowledge about the role of drug-induced synaptic plasticity in the mPFC. *In vitro* studies have shown that nicotine can increase the threshold for the induction of spike-timing-dependent potentiation at glutamatergic synapses onto mouse mPFC layer V pyramidal neurons by enhancing GABAergic inputs to these neurons (Couey et al., 2007). In contrast, 5 days after withdrawal from repeated noncontingent cocaine exposure (seven daily injections), the induction of LTP was facilitated at excitatory synapses onto rat mPFC layer V pyramidal neurons due to reduced GABA input (Lu et al., 2010). This was mediated by a brain-derived neurotrophic factor (BDNF)-induced reduction of surface expression of GABA_A_ receptors in the mPFC, leading to suppression of GABAergic inhibition. Although the behavioral relevance of this enhanced plasticity was not extensively investigated, there is some indirect evidence that the facilitation of LTP induction in the mPFC might contribute to behavioral sensitization after cocaine withdrawal (Lu et al., 2010).

Despite the reduction of mPFC LTP by acute application of nicotine, induction of synaptic potentiation in mPFC is facilitated by nicotine withdrawal, similar to cocaine. During and immediately following *in vivo* nicotine treatment, glutamatergic synaptic potentiation was reduced in the mPFC of adolescent rats, while this LTP was significantly increased in the mPFC of adult rats 5 weeks after they received nicotine treatment during adolescence (Goriounova and Mansvelder, 2012). Bidirectional effects of nicotine exposure during adolescence on synaptic potentiation are most likely mediated by bidirectional changes in synaptic levels of the inhibitory metabotropic glutamate receptor 2 (mGluR2). Following nicotine exposure during adolescence, protein levels of mGluR2 in rat mPFC synaptic membranes were increased on the first day of withdrawal, but decreased 5 weeks after nicotine exposure (Counotte et al., 2011). Using mGluR2 agonists, nicotine-induced enhancement of synaptic potentiation could be blocked, while mGluR2 antagonists facilitated synaptic potentiation (Goriounova and Mansvelder, 2012).

The short-term block of LTP induction is likely to also be caused by a transient increase in the expression of nAChRs containing α4 and β2 subunits in the mPFC acutely after nicotine exposure during adolescence, which is the mechanism behind the enhanced GABAergic inhibition that causes this block of LTP (Counotte et al., 2012). Interestingly, the increase in the expression of nAChRs containing α4 and β2 subunits in the rat mPFC was only found after nicotine exposure during adolescence, and not after nicotine exposure during adulthood (Counotte et al., 2012). Similarly, the study by Goriounova and Mansvelder (2012) demonstrated a difference between the effects of nicotine exposure during adolescence and during adulthood. Nicotine exposure during adulthood did not lead to lasting changes in LTP. In line with this, adolescent nicotine exposure, but not postadolescent nicotine exposure, leads to diminished attentional performance and increments in impulsive action 5 weeks after treatment in rats (Counotte et al., 2009, 2011). Nicotine exposure during adolescence elicits a lasting reduction in synaptic mGluR2 levels and enhancement of timing-dependent long-term potentiation in the rat mPFC that may cause cognitive deficits during adulthood. Altogether, these studies indicate that adolescence is a critical period of vulnerability for long-term effects of nicotine on the PFC.

After prolonged withdrawal, changes in glutamate receptor subunit distribution were seen in several studies. For example, increased expression of GluA2/3, GluA4, and GluN2B in the mPFC was reported after 2 weeks of abstinence from binge cocaine self-administration (Tang et al., 2004). However, no long-term effect on the synaptic membrane expression of glutamate receptor subunits in the mPFC was found after heroin self-administration (Van den Oever et al., 2008). It is possible that changes in glutamate receptor subunit distribution after prolonged withdrawal only arise after extended access to drugs. This is supported by the study by Ben-Shahar et al. (2009), who again found increased GluN2B expression after 2 weeks of abstinence, but only in rats that had had extended daily access to cocaine (Ben-Shahar et al., 2009).

Since the mPFC is thought to play an important role in cue-induced relapse to drug seeking, it was also investigated whether synaptic plasticity occurs in the mPFC at the moment of cue-induced relapse to drug seeking. Re-exposure to heroin-associated cues after 3 weeks of abstinence from heroin self-administration induced rapid synaptic depression in the mPFC of rats (Van den Oever et al., 2008). The synaptic membrane expression of AMPAR subunits GluA2 and GluA3 in the mPFC was reduced, and the expression of clathrin-coat assembly protein AP2m1, which is involved in clathrin-dependent endocytosis, was increased. This suggests that the re-exposure to heroin-associated cues induced clathrin-mediated endocytosis of GluA2/GluA3 AMPARs. Furthermore, a decreased AMPAR/NMDAR ratio and an increased rectification index were found in mPFC pyramidal neurons, confirming the occurrence of endocytosis of GluA2-containing AMPARs. In these assessments, dissociations between the dorsal and ventral mPFC were not made. Therefore, it is not known whether the cue-induced synaptic depression occurs in the entire mPFC or only in one of the subregions. However, the behavioral effect of blocking cue-induced endocytosis of GluA2-containing AMPARs was assessed separately for both mPFC subregions. Interestingly, blocking GluA2 endocytosis specifically in the ventral mPFC attenuated cue-induced relapse to heroin seeking, while blocking GluA2 endocytosis in the dorsal mPFC did not (Van den Oever et al., 2008). This suggests that the rapid endocytosis of GluA2-containg AMPARs and the resulting synaptic depression in the ventral mPFC are crucial for cue-induced relapse to heroin seeking. This fits with the idea that the ventral mPFC exerts inhibitory control over drug seeking. All in all, it can be hypothesized that cue-induced synaptic depression in the ventral mPFC impairs inhibitory control over drug seeking, thereby mediating relapse to heroin seeking.

## Drug-induced synaptic plasticity in the human brain

Drug-induced synaptic plasticity in the rodent mesocorticolimbic system contributes to the development and persistence of addiction. Do drugs of abuse actually induce synaptic plasticity in the human brain? Human imaging studies have shown that addiction in humans involves changes in activity in the same brain areas in which synaptic changes have been shown in animal models of addiction (Van den Oever et al., 2012). Although these studies did not directly investigate synaptic plasticity, they do show that addiction causes enduring adaptations in the mesocorticolimbic system in humans. Only few studies have directly investigated plasticity of glutamatergic synapses in the human brain, and, as a result, evidence of drug-induced synaptic plasticity is sparse. Experimentally, timing-dependent plasticity of motor-evoked potentials (MEPs) can be induced in human subjects by pairing peripheral nerve stimulation (PNS, analogous to presynaptic stimulation) with a transcranial magnetic stimulation (TMS) of the motor cortex (Stefan et al., 2000, 2002; Wolters et al., 2003; De Beaumont et al., 2012; Lu et al., 2012). Such paired associative stimulation (PAS) can induce both LTP-like increases and LTD-like decreases in MEP amplitude, depending on the relative timing of associated stimuli (Wolters et al., 2003, 2005; Thabit et al., 2010; De Beaumont et al., 2012; Lu et al., 2012; Conde et al., 2013; Koch et al., 2013). At the synaptic level, it was found that in surgically resected human brain tissue, cortical glutamatergic synapses can undergo both LTP and LTD throughout adulthood, in response to similar timing regimes as was used for PAS in human subjects (Testa-Silva et al., 2010, 2014; Verhoog et al., 2013). This plasticity of adult human synapses was dependent on postsynaptic NMDARs and L-type voltage-gated calcium channels. Grundey et al. (2012) used PAS in human subjects and found that during nicotine withdrawal, LTP-like synaptic plasticity induced by PAS was abolished in smokers, but that it could be rescued by administration of a nicotine patch (Grundey et al., 2012). This suggests that mechanisms of drug-induced synaptic plasticity may also play a role in the human brain. Clearly, more studies in human subjects and in surgically resected living human brain tissue are needed to verify that drug-induced synaptic changes found in rodents indeed also play a role in the human brain. Some successes have already been achieved though in trials in human addicts for treatments inspired by rodent research, as we will discuss below, supporting the idea that drug-induced synaptic changes and their behavioral consequences are indeed relevant for the development of addiction treatments.

## Novel targets for potential treatment strategies for addiction

Can drug-induced synaptic plasticity be a target for potential treatments of human addiction? We will discuss some ideas based on rodent addiction research why plasticity might actually offer promising targets. Since drug-induced synaptic plasticity in the VTA represents a first step in a cascade of synaptic changes throughout the mesocorticolimbic system, an early intervention at the level of the VTA might prevent synaptic changes in downstream brain regions such as the NAc. In rodents, cocaine-induced synaptic plasticity in the VTA can be reversed by activation of mGluR1 receptors (Bellone and Lüscher, 2006). Stimulation of mGluR1 receptors produces a form of mGluR-dependent LTD, which consists of a replacement of GluA2-lacking AMPARS with lower conductance GluA2-containing AMPARs (Mameli et al., 2007). This normalizes synaptic transmission. Furthermore, this type of mGluR-dependent LTD also reverses the cocaine-induced redistribution of NMDARs (Yuan et al., 2013). Selectively preventing synaptic plasticity in the VTA is sufficient to prevent subsequent synaptic plasticity in the NAc and to attenuate cue-induced cocaine seeking after prolonged withdrawal in mice (Mameli et al., 2009), suggesting that this strategy can be efficacious in the treatment of cocaine addiction in the rodent brain. In the NAc, mGluR1 positive modulators can also reverse the drug-induced synaptic plasticity that is seen after prolonged withdrawal (Wolf and Tseng, 2012). Therefore, mGluR1s might not only be a potential target to treat early stages of addiction, but might also act at later stages of the disease and attenuate incubation of drug craving. Whether mGluR1 activation actually reduces drug taking, drug craving and relapse in rodents still requires investigation. Also, whether mGluR1 activation normalizes synaptic strength in the VTA following synaptic plasticity induced by other drugs of abuse than cocaine is not known.

Another promising potential target is the acid-sensing ion channel 1A (ASIC1A), which inhibits cocaine-evoked synaptic plasticity in the NAc (Kreple et al., 2014). ASIC1A activity also attenuates addiction-related behavior. Overexpressing ASIC1A in rat NAc reduced cocaine self-administration while disruption of ASIC1A increased both morphine-CPP and cocaine-CPP in mice (Kreple et al., 2014). Enhancing ASIC1A function might therefore be beneficial in the treatment of addiction. ASIC1A function is thought to be reduced by the enzyme carbonic anhydrase IV (CA-IV), which has a role in the regulation of extracellular pH buffering in the brain (Kreple et al., 2014). Therefore, compounds such as CA-IV inhibitor acetazolamide that enhance ASIC1A function, might be a promising lead to follow.

A different strategy that might prove beneficial in the treatment of addiction is to rescue drug-induced deficits in NMDAR-dependent synaptic plasticity in the NAc, which contribute to inflexible, compulsive behaviors characteristic of addiction (D’Ascenzo et al., 2014). Since the deficits in NMDAR-dependent synaptic plasticity in the NAc are thought to be at least partially due to reduced D-serine levels, targeting D-serine signaling may hold promise for the treatment of drug addiction. D-serine levels can for example be restored by administration of an inhibitor of the enzyme D-amino acid oxidase, which degrades D-serine (D’Ascenzo et al., 2014). Thus far, the role of D-serine has only been studied in the context of cocaine addiction. Whether D-serine is also involved in addiction to other types of drugs of abuse is not known.

Many addiction treatments have limited success due to high relapse rates. Therapeutic strategies focusing on reducing relapse rates would therefore be promising. Given the role of the mPFC in control of relapse behavior, novel treatment strategies could be aimed at reducing activity in the dorsal mPFC or at enhancing glutamatergic transmission in the ventral mPFC. In particular, agents that inhibit cue-induced endocytosis of GluA2-containing AMPARs might be useful to prevent relapse. This was done in rat mPFC with a TAT peptide, TAT-GluR2_3γ_ (Van den Oever et al., 2008), which prevented GluA2-containing AMPAR endocytosis and reduced cue-induced heroin seeking in these rats.

Instead of reducing activity in the dorsal mPFC, another approach for preventing cue-induced relapse could be to reduce the impact of this activity at glutamatergic mPFC-to-NAc synapses in the NAc. During withdrawal from addictive drugs, extracellular glutamate levels in the NAc are reduced, which enhances the amount of glutamate that is released in mPFC-to-NAc synapses after exposure to drug-associated cues (Gipson et al., 2014). Furthermore, reduced GLT-1 levels diminish glutamate uptake from the synapse. Restoring GLT-1 and extracellular glutamate levels could be a way to prevent cue-induced relapse. One of the compounds that restores levels of GLT-1 and extracellular glutamate in the NAc is the antioxidant N-acetylcysteine (NAC; Gipson et al., 2014). NAC treatment has already been tested in human addicts, and was found to reduce drug craving and cocaine use (LaRowe et al., 2007; Mardikian et al., 2007).

## Conclusion and future directions

**Figure 1:**
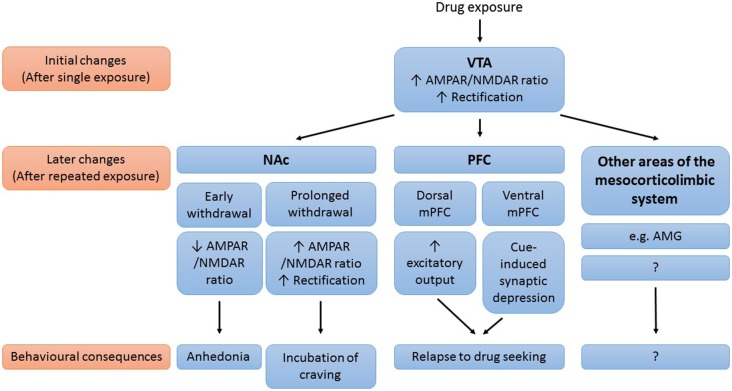
**Working model of the role of drug-induced synaptic plasticity in the mesocorticolimbic system in drug addiction**. Simplified schematic of the mesocorticolimbic brain areas known to be affected by drug-induced plasticity, important synaptic modifications that occur in these brain areas and the behavioral consequences of these modifications. AMG, amygdala; NAc, nucleus accumbens; (m)PFC, (medial) prefrontal cortex; VTA, ventral tegmental area.

Drug-induced synaptic modifications contribute to the development and persistence of addiction. Although our knowledge on drug-induced synaptic plasticity is far from complete, a picture is emerging in which glutamatergic synaptic plasticity in mesocorticolimbic areas plays an important role in drug addiction (Figure 1). Initially, exposure to an addictive drug induces synaptic changes in the VTA. In VTA DA neurons, drug exposure results in synaptic insertion of high conductance GluA2-lacking AMPARs in exchange for lower conductance GluA2-containing AMPARs. This drug-induced synaptic potentiation in the VTA subsequently triggers synaptic changes in downstream areas of the mesocorticolimbic system (such as the NAc, the PFC, and the amygdala) with further drug exposure. These synaptic adaptations then mediate many of the behavioral symptoms that characterize addiction. The decreased AMPAR/NMDAR ratio in the NAc during early withdrawal might mediate feelings of needing the drug to feel happy, by making neurons less sensitive to natural rewarding stimuli. The subsequent gradual potentiation of AMPAR transmission, the gradual maturation of silent synapses, and the reduced basal extracellular glutamate levels in the NAc all mediate the expression of incubation of cue-induced craving, by enhancing the responsiveness of NAc MSNs to drug-associated cues. Future studies in which synaptic modifications (and their behavioral consequences) are investigated in both NAc core and shell are needed to dissociate the role of NAc core and shell in this working model. The drug-induced synaptic adaptations in the PFC play an important role in relapse. Rapid cue-induced synaptic depression in the ventral mPFC impairs response inhibition upon exposure to drug-associated cues, thereby mediating relapse to drug seeking. Drug-induced neuroadaptations in the dorsal mPFC on the other hand act to enhance excitatory output from this subregion to NAc core, which drives drug seeking.

More research is needed to verify and extend this working model, since the synaptic changes that are induced by addictive drugs are numerous and complex. Several important questions remain unanswered. For instance, drug-evoked synaptic plasticity has been predominantly studied in the VTA and NAc, but our knowledge on synaptic changes in the mPFC and their behavioral consequences is still limited. In addition, what drug-induced neuroadaptations take place in other brain areas that play a role in addiction, such as the amygdala and habenula (Maroteaux and Mameli, 2012; Van den Oever et al., 2012; Lecca et al., 2014), where plasticity can also be very prominent? The majority of the studies discussed in this review specifically examined cocaine-induced synaptic plasticity. Do the findings generalize to other drugs of abuse? This is especially important since different types of drugs can have different effects on synaptic transmission (Wolf and Ferrario, 2010). Another unresolved issue is the neural basis of individual differences in vulnerability to drug addiction. Studies that compare effects of addictive drugs in animals that develop addiction-like behaviors with the effects of addictive drugs in animals that do not may contribute to finding the synaptic correlates of addiction vulnerability (Kasanetz et al., 2010; Abrahao et al., 2013). Finally, drugs of abuse can differentially affect subpopulations of neurons within a specific brain region (Wolf and Ferrario, 2010; Creed and Lüscher, 2013). It will be exciting to learn how heterogeneous responses of specific subpopulations of neurons to drugs and drug-associated cues in different mesocorticolimbic areas give rise to the various aspects of addiction.

## Conflict of interest statement

The authors declare that the research was conducted in the absence of any commercial or financial relationships that could be construed as a potential conflict of interest.
